# Fatores Associados à Alta Prevalência de Síndrome Metabólica em Vasculite Associada a Anticorpos Anticitoplasma de Neutrófilos

**DOI:** 10.36660/abc.20260076

**Published:** 2026-05-29

**Authors:** Marina Lamas Figueredo, Matheus Alves Amaral de Lima, Alexandre Moura dos Santos, Samuel Katsuyuki Shinjo

**Affiliations:** 1 Universidade de São Paulo Faculdade de Medicina FMUSP Disciplina de Reumatologia São Paulo SP Brasil Disciplina de Reumatologia, Faculdade de Medicina FMUSP, Universidade de São Paulo, São Paulo, SP – Brasil

**Keywords:** Doenças Cardiovasculares, Vasculite Associada a Anticorpo Anticitoplasma de Neutrófilos, Síndrome Metabólica, Vasculite Sistêmica

## Abstract

**Fundamento:**

A síndrome metabólica (SM) permanece incompletamente caracterizada na vasculite associada a anticorpos anticitoplasma de neutrófilos (VAA). Este estudo teve como objetivo determinar a prevalência de SM em pacientes com VAA e identificar fatores associados à sua presença.

**Métodos:**

Este estudo transversal, unicêntrico, incluiu 62 pacientes com VAA (42 com granulomatose com poliangiite, 15 com granulomatose eosinofílica com poliangiite e cinco com poliangiite microscópica) em acompanhamento regular em um hospital terciário de referência. Os pacientes foram pareados por idade e sexo com 53 controles. A SM foi definida de acordo com os critérios do *National Cholesterol Education Program Adult Treatment Panel III*. A significância estatística foi padronizada em p < 0,05.

**Resultados:**

A mediana de idade dos pacientes foi de 56,1 anos (intervalo interquartil, 45,6-65,5); 53,2% eram mulheres e 85,5% eram brancos. A prevalência de SM foi significativamente maior no grupo VAA do que nos controles (43,5% vs 13,2%; p = 0,001). Diabetes melito, hipertensão arterial sistêmica e história familiar de doença cardiovascular foram mais frequentes entre os pacientes com VAA, enquanto o índice de massa corporal (IMC) e a inatividade física não diferiram entre os grupos. Na análise multivariada, SM (*odds ratio* [OR], 4,52; intervalo de confiança de 95% [IC95%], 1,66-12,27; p = 0,003) e história familiar de doença cardiovascular (OR, 3,81; IC95%, 1,09-13,34; p < 0,05) estiveram independentemente associadas à VAA. Entre os pacientes com VAA, não foram observadas diferenças entre aqueles com e sem SM quanto ao subtipo de VAA, medidas antropométricas, atividade da doença, história familiar, inatividade física ou tratamento atual. Em análise multivariada restrita aos pacientes com VAA, a SM esteve independentemente associada ao sexo masculino (OR, 11,43; IC95%, 2,81-46,52; p = 0,001) e a maior IMC (OR, 1,13; IC95%, 1,02-1,25; p = 0,022).

**Conclusões:**

A SM apresentou alta prevalência entre pacientes com VAA e esteve associada ao sexo masculino e a maior IMC, contribuindo para um perfil de risco cardiometabólico desfavorável nesta coorte unicêntrica.

**Figure:**
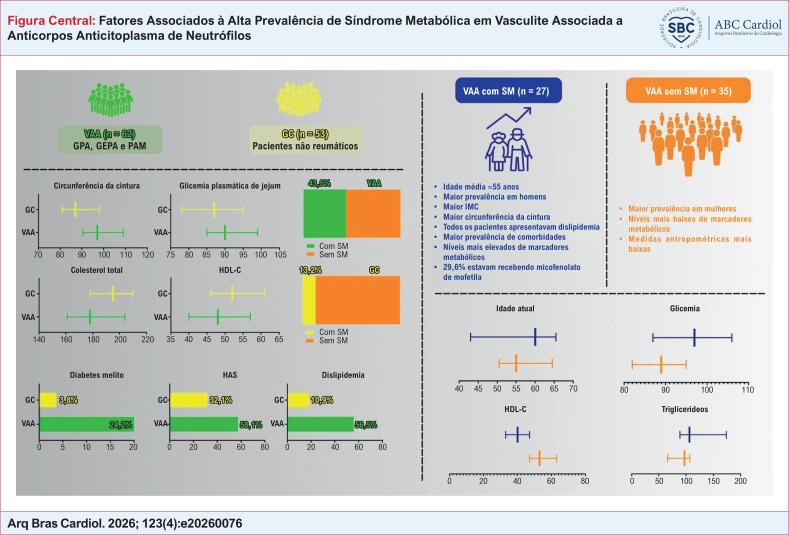
GEPA: granulomatose eosinofílica com poliangiite; GC: grupo controle; GPA: granulomatose com poliangiite; HAS: hipertensão arterial sistêmica; HDL-C: lipoproteína de alta densidade colesterol; IMC: índice de massa corporal; PAM: poliangiite microscópica; SM: síndrome metabólica; VAA: vasculite associada a anticorpos anticitoplasma de neutrófilos.

## Introdução

A vasculite associada a anticorpos (VAA) anticitoplasma de neutrófilos (ANCA) compreende um grupo raro de doenças autoimunes caracterizadas por inflamação necrosante de vasos sanguíneos de pequeno e médio calibre. A VAA inclui três subtipos: granulomatose com poliangiite (GPA), poliangiite microscópica (PAM) e granulomatose eosinofílica com poliangiite (GEPA).^1^ Essas condições estão comumente associadas à presença de ANCA circulantes em exames laboratoriais.^2^

A síndrome metabólica (SM) é definida pela presença de pelo menos três alterações metabólicas que, em conjunto, aumentam o risco de diabetes melito tipo 2 e doença cardiovascular (DCV).^3^ As evidências sobre SM na VAA permanecem limitadas. Estudos disponíveis indicam que pacientes com VAA apresentam níveis aumentados de proteína C-reativa^4^ e maior prevalência de SM em comparação com a população geral.^5^ Além disso, a SM parece ser um fator de risco independente para DCV na VAA,^6^ e a presença de mais de três componentes da SM foi identificada como um preditor independente de mortalidade entre pacientes com VAA e SM.^7^

Devido ao número limitado de estudos que avaliam a SM na VAA, este estudo teve como objetivo determinar a prevalência de SM em uma coorte de pacientes consecutivos com VAA em acompanhamento regular em um centro terciário de referência, utilizando uma amostra de conveniência, bem como comparar esses achados com os de um grupo controle (GC). Adicionalmente, buscou-se identificar fatores potencialmente associados à presença de SM nessa população, com ênfase principal na caracterização clínica e metabólica detalhada desta coorte unicêntrica, em vez de gerar estimativas epidemiológicas populacionais.

## Métodos

### Local e amostra do estudo

Este estudo transversal, unicêntrico, foi concebido principalmente como uma investigação clínica descritiva da prevalência de SM e de fatores de risco cardiometabólicos em pacientes com VAA em acompanhamento regular na Unidade de Vasculites de um hospital terciário de referência. O protocolo do estudo foi aprovado pelo comitê de ética em pesquisa com seres humanos local.

Todos os pacientes preencheram os critérios de classificação para VAA de 2022 do *American College of Rheumatology* (ACR)/*European Alliance of Associations for Rheumatology* (EULAR).^8–10^

### População do estudo e recrutamento

A coorte de VAA foi composta por pacientes prevalentes consecutivos em acompanhamento regular contínuo na Unidade de Vasculites durante o período do estudo, incluindo uma combinação de casos recentemente diagnosticados e de longa duração. Todos os indivíduos que atenderam aos critérios de elegibilidade foram incluídos, resultando em uma amostra de conveniência de 62 pacientes. Não foi realizado cálculo formal do tamanho amostral. A distribuição desigual dos subtipos de VAA (isto é, GPA, GEPA e PAM) reflete os padrões de encaminhamento e a subespecialização local na Unidade de Vasculites terciária, e não proporções populacionais, não tendo sido realizada amostragem estratificada por subtipo de VAA.

Os pacientes foram entrevistados durante consultas ambulatoriais de rotina. Foram coletados os seguintes dados: características demográficas (isto é, idade, sexo e etnia); variáveis relacionadas à doença (início dos sintomas, duração da doença [definida como o intervalo entre o diagnóstico e a visita do estudo], manifestações clínicas e laboratoriais, tratamentos, curso da doença, recaídas e complicações); histórico cardiovascular (acidente vascular cerebral prévio, infarto agudo do miocárdio [IAM] ou insuficiência cardíaca congestiva [ICC]); e histórico familiar de DCV, definido como IAM ocorrendo antes dos 55 anos no pai e/ou antes dos 65 anos na mãe.

Em relação à exposição a glicocorticoides, apenas a dose atual de prednisona no momento da visita do estudo foi sistematicamente registrada e analisada, uma vez que informações detalhadas sobre a exposição cumulativa ao longo da vida, especialmente tratamentos realizados antes do encaminhamento ao nosso centro, não estavam consistentemente disponíveis para todos os pacientes.

As medidas antropométricas incluíram peso, altura, índice de massa corporal (IMC) e composição corporal. A espessura das dobras cutâneas foi mensurada com adipômetro de acordo com o protocolo de Durnin e Womersley,^11^ incluindo dobras bicipital, tricipital, subescapular e suprailíaca. Todas as medidas foram padronizadas e realizadas pelo mesmo avaliador treinado no lado direito do corpo, com os participantes em posição ortostática. A densidade corporal foi estimada utilizando equações específicas para sexo e idade propostas por Durnin e Womersley,^11^ e a porcentagem de gordura corporal foi posteriormente calculada pela equação de Siri.^12^ Os valores de referência foram interpretados de acordo com sexo e idade, considerando a maior porcentagem fisiológica de gordura corporal em mulheres.^13,14^

Os componentes da SM foram avaliados com base em dados clínicos e laboratoriais segundo os critérios do *National Cholesterol Education Program Adult Treatment Panel III:*^15^ colesterol de lipoproteína de alta densidade (HDL-C) < 40 mg/dl em homens ou < 50 mg/dl em mulheres, ou uso de terapia hipolipemiante; triglicerídeos ≥ 150 mg/dl; glicemia de jejum ≥ 100 mg/dl; circunferência da cintura > 102 cm em homens ou > 88 cm em mulheres; e pressão arterial ≥ 130/85 mmHg ou uso de medicação anti-hipertensiva. O diagnóstico de SM exigiu a presença de pelo menos três desses componentes.

O estado funcional foi avaliado pelo *Health Assessment Questionnaire* (HAQ),^16^ que varia de 0,00 (sem incapacidade) a 3,00 (incapacidade grave). A atividade física foi avaliada pelo *International Physical Activity Questionnaire-Short Form* (IPAQ-SF),^17^ que mensura a frequência e a duração de caminhada, atividades físicas de intensidade moderada e vigorosa, bem como o tempo sentado durante dias de semana e finais de semana.

### Grupo controle

O GC foi composto por voluntários sem doenças reumáticas, recrutados entre funcionários do hospital e acompanhantes de pacientes que concordaram em participar e atenderam aos critérios de inclusão. Os controles foram pareados por frequência com os pacientes quanto à distribuição de sexo e idade. Um total de 53 controles completou a avaliação clínica e laboratorial completa durante o período do estudo, resultando em um grupo ligeiramente menor que a coorte de VAA. Essa estratégia pragmática de recrutamento, e a consequente diferença no tamanho dos grupos, refletem limitações práticas inerentes ao recrutamento baseado em voluntários, e não uma escolha metodológica deliberada. Reconhece-se que essa abordagem pode introduzir viés de seleção e que o GC pode não ser representativo da população geral.

Nos controles, foram coletados dados demográficos, bem como informações sobre DCV e fatores de risco associados, incluindo acidente vascular cerebral prévio, IAM ou ICC, além de comorbidades como hipertensão arterial sistêmica (HAS), diabetes melito, dislipidemia e hipotireoidismo. Foram obtidas medidas antropométricas, incluindo IMC e circunferência da cintura. A inatividade física foi definida como a ausência de qualquer atividade física com duração mínima de 10 minutos contínuos por semana, conforme avaliado pelo IPAQ-SF. Os dados laboratoriais incluíram glicemia de jejum, colesterol total, triglicerídeos, colesterol de lipoproteína de baixa densidade (LDL-C) e HDL-C.

Todos os dados foram armazenados no REDCap. Informações sensíveis foram anonimizadas em conformidade com a Lei Geral de Proteção de Dados.

### Análise estatística

As variáveis contínuas são apresentadas como média ± desvio padrão para dados com distribuição normal ou como mediana e intervalo interquartil (IIQ, percentis 25-75) para dados com distribuição não normal. As variáveis categóricas são expressas como números absolutos e porcentagens. A distribuição dos dados foi avaliada pelo teste de Shapiro-Wilk.

As comparações entre grupos independentes foram realizadas utilizando o teste *t* de Student não pareado para variáveis contínuas com distribuição normal ou o teste *U* de Mann-Whitney para variáveis com distribuição não normal. As variáveis categóricas foram comparadas por meio do teste do qui-quadrado ou do teste exato de Fisher, conforme apropriado.

As associações foram avaliadas por regressão logística binária e são apresentadas como *odds ratios* (OR) com intervalos de confiança de 95% (IC95%). Um valor de p bicaudal < 0,05 foi considerado estatisticamente significativo. As análises estatísticas foram realizadas no GraphPad Prism, versão 8.0.2 (GraphPad Software, San Diego, CA, EUA).

## Resultados

O estudo incluiu 62 pacientes com VAA e 53 no GC (Tabela 1, Figura Central). As distribuições de idade, sexo e etnia foram semelhantes entre os grupos. A mediana de idade no início da doença entre os pacientes foi de 42,0 anos (IIQ, 27,0-53,5). A adiposidade global, refletida pelo IMC, não diferiu entre pacientes e controles; entretanto, a adiposidade abdominal (central) foi significativamente maior no grupo VAA, conforme indicado pela maior circunferência da cintura (Tabela 1). A combinação de IMC semelhante e maior circunferência da cintura em pacientes com VAA sugere predominância de obesidade central, a qual está mais fortemente associada ao risco cardiometabólico do que a obesidade generalizada.

**Tabela 1:** Dados demográficos, clínicos, de estilo de vida, comorbidades e laboratoriais de pacientes com VAA e GC

Variáveis	VAA (n = 62)	GC (n = 53)	Valor de p
**Idade atual, anos**	56,1 (45,6-65,5)	51,0 (47,0-57,0)	0,115
**Idade ao diagnóstico, anos**	42,0 (27,0-53,5)	—	—
**Sexo (masculino)**	29 (46,8)	20 (37,7)	0,329
**Etnia (branca)**	53 (85,5)	46 (86,8)	0,840
**IMC, kg/m^2^**	28,1 (23,7-32,5)	25,5 (23,0-30,7)	0,174
**Circunferência da cintura, cm**	97,0 (90,5-109,0)	87,0 (81,0-98,0)	0,001
**HAS**	36 (58,1)	17 (32,1)	0,005
**Diabetes melito**	15 (24,2)	2 (3,8)	0,003
**Dislipidemia**	35 (56,5)	10 (18,9)	< 0,001
**AVE isquêmico**	1 (1,6)	0	> 0,999
**IAM**	2 (3,2)	0	0,499
**ICC**	1 (1,6)	0	> 0,999
**Hipotireoidismo**	7 (11,3)	5 (9,4)	> 0,999
**História familiar de DCV**	14 (22,6)	4 (7,5)	0,038
**Inatividade física**	23 (37,1)	18 (34,0)	0,133
**Glicemia de jejum, mg/dl**	90 (85-99)	87 (78-95)	0,001
**Triglicerídeos, mg/dl**	101 (76-133)	100 (74-122)	0,545
**Colesterol total, mg/dl**	178 (161-204)	195 (178-210)	0,013
**HDL-C, mg/dl**	48 (40-57)	52 (46-61)	0,019
**LDL-C, mg/dl**	112 (100-127)	117 (101-130)	0,511
**SM**	27 (43,5)	7 (13,2)	0,001

Os resultados são expressos como porcentagem (%) ou mediana (IIQ, percentis 25-75). AVE: acidente vascular encefálico; DCV: doença cardiovascular; GC: grupo controle; HAS: hipertensão arterial sistêmica; HDL-C: colesterol de lipoproteína de alta densidade; IAM: infarto agudo do miocárdio; ICC: insuficiência cardíaca congestiva; IIQ: intervalo interquartil; IMC: índice de massa corporal; LDL-C: colesterol de lipoproteína de baixa densidade; SM: síndrome metabólica; VAA: vasculite associada a anticorpos anticitoplasma de neutrófilos.

As comorbidades foram, de modo geral, comparáveis entre os grupos, exceto por maior prevalência de HAS, diabetes melito e dislipidemia entre os pacientes. Não foram relatados casos de acidente vascular cerebral isquêmico, IAM ou ICC no GC. Em contraste, entre os pacientes com VAA, foram registrados dois casos de IAM (3,2%), um de acidente vascular cerebral isquêmico (1,6%) e um episódio de ICC (1,6%). A história familiar positiva para DCV foi mais frequente nos pacientes do que nos controles (22,6% vs 7,5%; p = 0,038).

A análise laboratorial demonstrou níveis mais elevados de glicemia de jejum e níveis mais baixos de colesterol total nos pacientes em comparação com os controles. Os níveis de triglicerídeos e LDL-C foram semelhantes entre os grupos, enquanto os níveis de HDL-C foram menores no grupo VAA.

Na análise multivariada, tanto a história familiar de DCV (OR, 3,81; IC95%, 1,09-12,34; p = 0,036) quanto a SM (OR, 4,52; IC95%, 1,66-12,27; p = 0,003) permaneceram independentemente associadas à VAA.

Entre os pacientes com VAA, 27 apresentavam SM e 35 não apresentavam. O subtipo de VAA (isto é, GPA, GEPA ou PAM), o IMC global, a porcentagem de gordura corporal, a incidência de eventos cardiovasculares, a inatividade física, o hipotireoidismo, o diabetes melito, a história familiar de DCV e os níveis de colesterol total e LDL-C foram semelhantes entre pacientes com e sem SM. Na análise univariada, pacientes com SM apresentaram maior circunferência da cintura, maior prevalência do sexo masculino, HAS e dislipidemia, além de níveis mais elevados de glicemia de jejum e triglicerídeos e níveis mais baixos de HDL-C (Tabela 2). A duração da doença, refletida pelo tempo de acompanhamento na Tabela 2, não diferiu significativamente entre os grupos.

**Tabela 2:** Dados demográficos, clínicos, de estilo de vida, comorbidades, características da doença e laboratoriais de pacientes com VAA de acordo com o status de SM

Variáveis	VAA com SM (n = 27)	VAA sem SM (n = 35)	valor de p
**GPA**	19 (70,4)	23 (65,7)	0,697
**GEPA**	5 (18,5)	10 (28,6)	0,359
**PAM**	3 (11,1)	2 (5,7)	0,645
**Idade atual, anos**	55,0 (50,5-64,5)	60,0 (43,0-65,5)	0,042
**Idade ao diagnóstico, anos**	45,0 (37,5-52,8)	47,0 (32,0-57,5)	0,828
**Seguimento, meses**	67,0 (21,5-135,5)	49,0 (21,0-91,5)	0,575
**Sexo (masculino)**	19 (70,4)	9 (25,7)	0,001
**Etnia (branca)**	21 (77,8)	32 (91,4)	0,160
**IMC, kg/m^2^**	29,7 (26,7-32,8)	26,2 (22,6-32,4)	0,042
**Circunferência da cintura, cm**	104,0 (97,0-110,0)	94,0 (84,5-103,0)	0,013
**Gordura corporal, %**	34,1 (28,5-39,2)	29,9 (24,1-34,2)	0,070
**HAS**	21 (77,8)	15 (42,9)	0,001
**Diabetes melito**	9 (33,3)	6 (17,1)	0,140
**Dislipidemia**	27 (100)	11 (31,4)	< 0,001
**AVE isquêmico**	0	1 (2,9)	> 0,999
**IAM**	1 (3,7)	1 (2,9)	> 0,999
**ICC**	0	1 (2,9)	> 0,999
**Hipotireoidismo**	2 (7,4)	5 (14,3)	0,455
**História familiar de DCV**	7 (25,9)	9 (25,7)	0,984
**HAQ**	0,5 (0,0-0,75)	0,3 (0,0-1,6)	0,520
**Inatividade física**	11 (40,7)	12 (34,3)	0,602
**Glicemia de jejum, mg/dl**	97 (87-106)	89 (82-95)	0,008
**Triglicerídeos, mg/dl**	106 (89-174)	97 (66-107)	0,027
**Colesterol total, mg/dl**	171 (161-196)	182 (163-204)	0,489
**HDL-C, mg/dl**	40 (33-47)	53 (47-63)	< 0,001
**LDL-C, mg/dl**	113 (101-127)	106 (85-125)	0,438
**Doença ativa**	11 (40,7)	12 (34,3)	0,602
**Uso de prednisona**	8 (29,6)	13 (37,1)	0,535
**Dose de prednisona, mg/dia**	7,5 (5,0-20,0)	10,0 (5,0-20,0)	0,654
**Terapia IS/IB**	21 (77,8)	27 (77,1)	0,953
**Azatioprina**	8 (29,6)	15 (42,9)	0,285
**Metotrexato**	6 (22,2)	7 (20,0)	0,831
**Micofenolato de mofetila**	8 (29,6)	3 (8,6)	0,045
**Ciclofosfamida**	2 (7,4)	0	0,186
**Rituximabe**	5 (18,5)	11 (31,4)	0,294

Os resultados são expressos como porcentagem (%) ou mediana (IIQ, percentis 25-75). AVE: acidente vascular encefálico; DCV: doença cardiovascular; GEPA: granulomatose eosinofílica com poliangiite; GPA: granulomatose com poliangiite; HAQ: Health Assessment Questionnaire; HAS: hipertensão arterial sistêmica; HDL-C: colesterol de lipoproteína de alta densidade; IAM: infarto agudo do miocárdio; ICC: insuficiência cardíaca congestiva; IIQ: intervalo interquartil; IMC: índice de massa corporal; IS/IB: imunossupressores/imunobiológicos; LDL-C: colesterol de lipoproteína de baixa densidade; PAM: poliangiite microscópica; SM: síndrome metabólica; VAA: vasculite associada a anticorpos anticitoplasma de neutrófilos.

A atividade da doença e o uso de prednisona (incluindo a dose mediana), agentes imunossupressores ou terapias biológicas foram semelhantes entre os grupos. No entanto, o micofenolato de mofetila foi utilizado com maior frequência entre os pacientes com SM (p = 0,045).

Na análise multivariada, apenas o sexo masculino (OR, 11,43; IC95%, 2,81-46,52; p = 0,001) e maior IMC (OR, 1,13; IC95%, 1,02-1,25; p = 0,022) permaneceram independentemente associados à presença de SM em pacientes com VAA.

## Discussão

Este estudo demonstrou alta prevalência de SM entre pacientes com VAA, juntamente com uma associação independente entre VAA, SM e história familiar de DCV (Figura Central). Na análise multivariada restrita aos pacientes com VAA, a SM esteve independentemente associada ao sexo masculino e a maior IMC. Entre os pacientes com VAA, idade mais jovem e maior IMC estiveram independentemente associadas à SM. Um ponto forte deste estudo é o uso dos critérios de classificação mais recentes do ACR/EULAR para VAA, o que aumenta a acurácia diagnóstica e a homogeneidade da coorte.^8–10^ Além disso, este trabalho fornece uma caracterização clínica e metabólica detalhada de uma coorte unicêntrica de pacientes consecutivos com VAA em acompanhamento regular, utilizando uma definição contemporânea de SM.

Uma alta prevalência de SM tem sido relatada em diversas doenças reumáticas autoimunes sistêmicas, incluindo lúpus eritematoso sistêmico, esclerose sistêmica, artrite reumatoide, espondiloartrites e miopatias autoimunes.^18–23^ Na VAA, estudos prévios relataram prevalências variando de 43% a 51,4%.^4–7^ Nossos achados são consistentes com os de Smits et al.^4^ e Lee et al.^6^ A menor prevalência observada entre os controles pode refletir diferenças nas características populacionais ou na metodologia. A maioria dos estudos prévios em VAA foi retrospectiva,^4,6,7^ e o único estudo transversal incluiu uma amostra pequena.^5^ Estudos anteriores também utilizaram critérios da European Medicines Agency ou de Chapel Hill, enquanto o presente estudo aplicou as definições atualizadas do ACR/EULAR.^8–10^

Em contraste com relatos prévios que identificam a idade avançada como fator de risco clássico para SM na população geral, os pacientes com SM nesta coorte eram ligeiramente mais jovens do que aqueles sem SM. Esse achado pode refletir exposição mais precoce e mais intensa a glicocorticoides e terapias imunossupressoras em pacientes mais jovens, além de vieses de sobrevida e encaminhamento, conforme descrito em outras vasculites, como a arterite de Takayasu.^24^ Pacientes mais jovens com doença mais grave ou ativa podem ser acompanhados com maior frequência em centros terciários, enquanto pacientes mais idosos, com maior carga cardiometabólica e eventos cardiovasculares prévios, podem estar sub-representados nesta amostra transversal. A maioria dos pacientes com SM era do sexo masculino, em concordância com Park et al.^7^ e com dados recentes que indicam aumento da prevalência de SM entre homens mais jovens na população geral.^25,26^

Maior IMC esteve independentemente associado à SM, em concordância com achados prévios.^6^ Embora o IMC não tenha diferido entre pacientes com VAA e controles, a circunferência da cintura foi maior nos pacientes, particularmente entre aqueles com SM, evidenciando adiposidade central. A combinação de IMC semelhante e maior circunferência da cintura em pacientes com VAA sugere predominância de obesidade central (abdominal), a qual está mais fortemente associada ao risco cardiometabólico do que a obesidade generalizada. A porcentagem de gordura corporal não diferiu entre os grupos; a adipometria foi considerada apropriada dada sua viabilidade e a baixa prevalência de obesidade.

Fatores de risco cardiovasculares foram mais frequentes entre pacientes com VAA, especialmente entre aqueles com SM. HAS e diabetes melito foram mais comuns em pacientes com VAA do que em controles, enquanto os níveis de triglicerídeos foram maiores em pacientes com SM, mas semelhantes entre VAA e controles. Os níveis de colesterol total e HDL-C foram menores em pacientes com VAA, em concordância com alterações lipídicas relacionadas à inflamação.^6^ Os níveis de LDL-C não diferiram, divergindo de alguns relatos prévios.^6^ Os níveis de glicemia de jejum estavam elevados tanto em pacientes com VAA quanto naqueles com SM. A história familiar de DCV foi mais frequente em pacientes com VAA, enquanto a inatividade física e os escores do HAQ foram semelhantes entre os grupos. Na análise multivariada incluindo pacientes e controles, SM e história familiar positiva para DCV permaneceram independentemente associadas à VAA, reforçando o perfil cardiometabólico desfavorável desta coorte.

Não foi observada associação entre SM e subtipo de VAA,^5,6^ e o tratamento farmacológico foi, em grande parte, comparável entre os grupos, exceto pelo uso mais frequente de micofenolato de mofetila entre pacientes com SM. Esse achado inesperado deve ser interpretado com cautela. O micofenolato de mofetila é frequentemente escolhido para pacientes com envolvimento orgânico específico ou comorbidades pré-existentes que desestimulam o uso de outros agentes imunossupressores. Portanto, a maior frequência de uso de micofenolato entre pacientes com SM provavelmente reflete confundimento por indicação e preferências médicas, e não um efeito causal direto do fármaco sobre parâmetros metabólicos. Até onde se sabe, não há evidências robustas que sustentem efeito diabetogênico ou dislipidêmico do micofenolato de mofetila, sendo necessários estudos adicionais para esclarecer essa associação.

Este estudo apresenta várias limitações. Primeiro, seu delineamento transversal impede inferência causal e não permite determinar se a SM precedeu o início da VAA ou se se desenvolveu como consequência da doença e de seu tratamento. Segundo, trata-se de um estudo unicêntrico baseado em uma amostra de conveniência de casos prevalentes consecutivos em acompanhamento regular em uma Unidade de Vasculites terciária, o que introduz viés de seleção e limita a generalização. Tanto a coorte de VAA quanto o GC, recrutado entre funcionários do hospital e acompanhantes de pacientes, provavelmente não são representativos das populações mais amplas de VAA ou geral. Assim, os achados devem ser interpretados como descritivos desta coorte clínica específica, e não como estimativas populacionais. Terceiro, o tamanho amostral relativamente pequeno reduz o poder estatístico e limita a inclusão de múltiplos confundidores clinicamente relevantes (como envolvimento orgânico detalhado e duração da doença) nos modelos multivariados. Quarto, embora o uso atual de prednisona e sua dose tenham sido sistematicamente registrados, informações detalhadas sobre a exposição cumulativa ao longo da vida, especialmente antes do encaminhamento ao nosso centro, não estavam consistentemente disponíveis, o que limita a avaliação de seu impacto sobre os componentes da SM. Por fim, eventos cardiovasculares incidentes após o diagnóstico de SM não foram avaliados, uma vez que o seguimento longitudinal estava além do escopo desta análise transversal.

Em conjunto, essas limitações indicam que inferências causais ou epidemiológicas robustas devem ser feitas com cautela. Estudos prospectivos, longitudinais e multicêntricos, com amostras maiores e coleta sistemática de exposições terapêuticas cumulativas e variáveis relacionadas à doença, são necessários para esclarecer a relação temporal entre VAA, SM e desfechos cardiovasculares, incluindo a incidência de novos eventos cardiovasculares após o diagnóstico de SM nessa população.

## Conclusões

A SM apresenta alta prevalência entre pacientes com VAA nesta coorte unicêntrica de conveniência e está associada ao sexo masculino e a maior IMC, contribuindo para um perfil de risco cardiovascular desfavorável. Esses achados destacam a necessidade de rastreamento sistemático e manejo agressivo dos componentes da SM em pacientes com VAA, em conjunto com o controle ideal da atividade da vasculite. A identificação precoce e o tratamento dos fatores de risco cardiometabólicos, associados a intervenções no estilo de vida, podem reduzir a morbidade cardiovascular e melhorar os desfechos em longo prazo, embora essa hipótese ainda necessite de confirmação em estudos prospectivos longitudinais.

## Data Availability

Todo o conjunto de dados que dá suporte aos resultados deste estudo está disponível mediante solicitação ao autor correspondente
