# Supplementary material for: Fatores Associados à Alta Prevalência de Síndrome Metabólica em Vasculite Associada a Anticorpos Anticitoplasma de Neutrófilos
Source: Arq Bras Cardiol. 2026 May 29;123(4):e20260076. [Article in Portuguese] doi: 10.36660/abc.20260076 (PMC13398801; doi:10.36660/abc.20260076)
Supplement: Translation

# Translation: Factors Associated With the High Prevalence of Metabolic Syndrome in Antineutrophil Cytoplasmic Antibody-Associated Vasculitis

**DOI:** 10.36660/abc.20260076i

**Authors:** Marina Lamas Figueredo, Matheus Alves Amaral de Lima, Alexandre Moura dos Santos, Samuel Katsuyuki Shinjo

**Affiliations:** 1 São Paulo SP Brazil Disciplina de Reumatologia, Faculdade de Medicina FMUSP, Universidade de São Paulo, São Paulo, SP – Brazil

**Keywords:** Cardiovascular Diseases, Anti-Neutrophil Cytoplasmic Antibody-Associated Vasculitis, Metabolic Syndrome, Systemic Vasculitis

## Abstract

**Background:**

Metabolic syndrome (MetS) remains incompletely characterized in antineutrophil cytoplasmic antibody-associated vasculitis (AAV). This study aimed to determine the prevalence of MetS in patients with AAV and to identify factors associated with its presence.

**Methods:**

This single-center, cross-sectional study included 62 patients with AAV (42 with granulomatosis with polyangiitis, 15 with eosinophilic granulomatosis with polyangiitis, and five with microscopic polyangiitis) who were under regular follow-up at a tertiary referral hospital. Patients were matched by age and sex with 53 controls. MetS was defined according to the National Cholesterol Education Program Adult Treatment Panel III criteria. Statistical significance was set at p < 0.05.

**Results:**

The median age of the patients was 56.1 years (interquartile range, 45.6-65.5); 53.2% were women, and 85.5% were White. The prevalence of MetS was significantly higher in the AAV group than in controls (43.5% vs 13.2%; p = 0.001). Diabetes mellitus, hypertension, and a family history of cardiovascular disease were more frequent among patients with AAV, whereas body mass index (BMI) and physical inactivity did not differ between groups. In multivariable analysis, MetS (odds ratio [OR], 4.52; 95%CI, 1.66-12.27; p = 0.003) and a family history of cardiovascular disease (OR, 3.81; 95%CI, 1.09-13.34; p < 0.05) were independently associated with AAV. Among patients with AAV, no differences were observed between those with and without MetS in terms of subtype of AAV, anthropometric measures, disease activity, family history, physical inactivity, or current treatment. In a multivariable analysis restricted to patients with AAV, MetS was independently associated with male sex (OR, 11.43; 95%CI, 2.81-46.52; p = 0.001) and higher BMI (OR, 1.13; 95%CI, 1.02-1.25; p = 0.022).

**Conclusions:**

MetS was highly prevalent among patients with AAV and was associated with male sex and higher BMI, contributing to an unfavorable cardiometabolic risk profile in this single-center cohort.

## Introduction

Antineutrophil cytoplasmic antibody (ANCA)-associated vasculitis (AAV) comprises a rare group of autoimmune disorders characterized by necrotizing inflammation of small- and medium-sized blood vessels. AAV includes three subtypes: granulomatosis with polyangiitis (GPA), microscopic polyangiitis (MPA), and eosinophilic granulomatosis with polyangiitis (EGPA).^1^ These conditions are commonly associated with circulating anti-ANCA on laboratory testing.^2^

Metabolic syndrome (MetS) is defined by the presence of at least three metabolic abnormalities that collectively increase the risk of type 2 diabetes mellitus and cardiovascular disease (CVD).^3^ Evidence on MetS in AAV remains limited. Available studies indicate that patients with AAV exhibit increased C-reactive protein levels^4^ and a higher prevalence of MetS compared with the overall population.^5^ Furthermore, MetS appears to be an independent risk factor for CVD in AAV,^6^ and the presence of more than three MetS components has been identified as an independent predictor of mortality among patients with AAV and MetS.^7^

Because of the limited number of studies evaluating MetS in AAV, this study aimed to determine the prevalence of MetS in a cohort of consecutive patients with AAV under regular follow-up at a tertiary referral center by using a convenience sample as well as to compare these findings with those of a control group (CTR). In addition, we sought to identify factors potentially associated with the presence of MetS in this population, with a primary emphasis on providing a detailed clinical and metabolic characterization of this single-center cohort rather than generating population-level epidemiological estimates.

**Figure:** AAV: antineutrophil cytoplasmic antibody-associated vasculitis; BMI: body mass index; CTR: control group; EGPA: eosinophilic granulomatosis with polyangiitis; GPA: granulomatosis with polyangiitis; HDL-C: high-density lipoprotein cholesterol; MetS: metabolic syndrome; MPA: microscopic polyangiitis.

## Methods

### Study setting and sample

This single-center, cross-sectional study was primarily designed as a descriptive clinical investigation of the prevalence of MetS and cardiometabolic risk factors in patients with AAV under regular follow-up at the Vasculitis Unit of a tertiary referral hospital. The study protocol was approved by the local human research ethics committee.

All patients fulfilled the 2022 American College of Rheumatology (ACR)/European Alliance of Associations for Rheumatology (EULAR) AAV classification criteria.^8–10^

### Study population and recruitment

The AAV cohort comprised consecutive prevalent patients undergoing ongoing regular follow-up at the Vasculitis Unit during the study period, including a mixture of recently diagnosed and long-standing cases. All individuals meeting the eligibility criteria were included, resulting in a convenience sample of 62 patients. No formal sample size calculation was performed. The uneven distribution of subtypes of AAV (i.e., GPA, EGPA, and MPA) reflects referral patterns and local subspecialization at our tertiary Vasculitis Unit rather than population-based proportions, and no stratified sampling by subtype of AAV was conducted.

Patients were interviewed during routine outpatient visits. The following data were collected: demographic characteristics (i.e., age, sex, and ethnicity); disease-related variables (symptom onset, disease duration [defined as the interval between diagnosis and the study visit], clinical and laboratory manifestations, treatments, disease course, relapses, and complications); cardiovascular history (prior stroke, acute myocardial infarction [AMI], or congestive heart failure [CHF]); and family history of CVD, defined as AMI occurring before 55 years of age in the father and/or before 65 years of age in the mother.

Regarding exposure to glucocorticoids, only the current prednisone dose at the time of the study visit was systematically recorded and analyzed, as detailed information on lifetime cumulative exposure to glucocorticoids, particularly treatment administered before referral to our center, was not consistently available across all patients.

Anthropometric measurements included weight, height, body mass index (BMI), and body composition. Skinfold thickness was measured using a skinfold caliper according to the Durnin and Womersley protocol,^11^ including biceps, triceps, subscapular, and suprailiac skinfolds. All measurements were standardized and performed by the same trained examiner on the right side of the body, with participants in an upright position. Body density was estimated using sex- and age-specific equations proposed by Durnin and Womersley,^11^ and body fat (BF) percentage was subsequently calculated using the Siri equation.^12^ Reference values were interpreted according to sex and age, acknowledging the physiologically higher BF percentage in women.^13,14^

MetS components were assessed using clinical and laboratory data based on the National Cholesterol Education Program Adult Treatment Panel III criteria ^15^: high-density lipoprotein cholesterol (HDL-C) < 40 mg/dL in men or < 50 mg/dL in women, or use of lipid-lowering therapy; triglycerides ≥ 150 mg/dL; fasting glucose ≥ 100 mg/dL; waist circumference > 102 cm in men or > 88 cm in women; and blood pressure ≥ 130/85 mmHg or use of antihypertensive medication. The MetS diagnosis required the presence of at least three of these components.

Functional status was assessed using the Health Assessment Questionnaire (HAQ),^16^ which ranges from 0.00 (no disability) to 3.00 (severe disability). Physical activity was evaluated using the International Physical Activity Questionnaire-Short Form (IPAQ-SF),^17^ which assesses the frequency and duration of walking, moderate- and vigorous-intensity physical activity, and sitting time during weekdays and weekends.

### Control group

The CTR consisted of volunteers without rheumatic diseases, recruited from hospital staff and patient companions who agreed to participate and met the inclusion criteria. Controls were frequency-matched to patients by sex and age distribution. A total of 53 controls completed the full clinical and laboratory assessment during the study period, resulting in a slightly smaller group than the AAV cohort. This pragmatic recruitment strategy, and the resulting difference in group sizes, reflect practical constraints inherent to volunteer-based recruitment rather than a deliberate methodological choice. We acknowledge that this approach may introduce selection bias and that the CTR may not be representative of the general population.

In controls, demographic data were collected along with information on CVD and associated risk factors, including prior stroke, AMI, or CHF as well as comorbidities such as hypertension, diabetes mellitus, dyslipidemia, and hypothyroidism. Anthropometric measurements, including BMI and waist circumference, were obtained. Physical inactivity was defined as the absence of any physical activity lasting at least 10 continuous minutes per week, as assessed by the IPAQ-SF. Laboratory data included fasting glucose, total cholesterol, triglycerides, low-density lipoprotein cholesterol (LDL-C), and HDL-C.

All data were stored in REDCap, and sensitive information was anonymized in accordance with the Brazilian General Data Protection Law.

### Statistical analysis

Continuous variables are presented as mean ± standard deviation for normally distributed data or as median and interquartile range (IQR, 25th-75th percentile) for non-normally distributed data. Categorical variables are expressed as absolute numbers and percentages. Data distribution was assessed using the Shapiro-Wilk test.

Comparisons between independent groups were performed using the unpaired Student's *t* test for normally distributed continuous variables or the Mann-Whitney *U* test for non-normally distributed variables. Categorical variables were compared using the chi-square test or Fisher's exact test, as appropriate.

Associations were evaluated using binary logistic regression and are reported as odds ratios (ORs) with 95%CI. A two-sided p-value < 0.05 was considered statistically significant. Statistical analyses were performed using GraphPad Prism, version 8.0.2 (GraphPad Software, San Diego, CA, USA).

## Results

The study included 62 patients with AAV and 53 in the CTR (Table 1, Central Illustration). Age, sex, and ethnicity distributions were similar between groups. The median age at disease onset among patients was 42.0 years (IQR, 27.0-53.5). Overall adiposity, as reflected by BMI, did not differ between patients and controls; however, abdominal (central) adiposity was significantly higher in the AAV group, as indicated by greater waist circumference (Table 1). The combination of similar BMI and higher waist circumference in patients with AAV suggests a predominance of central obesity, which is more strongly associated with cardiometabolic risk than generalized obesity.

**Table 1:** Demographic, clinical, lifestyle, comorbidity, and laboratory data of patients with AAV and CTR

Variables	AAV (n = 62)	CTR (n = 53)	p-value
**Current age, years**	56.1 (45.6-65.5)	51.0 (47.0-57.0)	0.115
**Age at diagnosis, years**	42.0 (27.0-53.5)	—	—
**Sex (male)**	29 (46.8)	20 (37.7)	0.329
**Ethnicity (White)**	53 (85.5)	46 (86.8)	0.840
**BMI, kg/m^2^**	28.1 (23.7-32.5)	25.5 (23.0-30.7)	0.174
**Waist circumference, cm**	97.0 (90.5-109.0)	87.0 (81.0-98.0)	0.001
**Hypertension**	36 (58.1)	17 (32.1)	0.005
**Diabetes mellitus**	15 (24.2)	2 (3.8)	0.003
**Dyslipidemia**	35 (56.5)	10 (18.9)	< 0.001
**Ischemic stroke**	1 (1.6)	0	> 0.999
**AMI**	2 (3.2)	0	0.499
**CHF**	1 (1.6)	0	> 0.999
**Hypothyroidism**	7 (11.3)	5 (9.4)	> 0.999
**Family history of CVD**	14 (22.6)	4 (7.5)	0.038
**Physical inactivity**	23 (37.1)	18 (34.0)	0.133
**Fasting blood glucose, mg/dL**	90 (85-99)	87 (78-95)	0.001
**Triglycerides, mg/dL**	101 (76-133)	100 (74-122)	0.545
**Total cholesterol, mg/dL**	178 (161-204)	195 (178-210)	0.013
**HDL-C, mg/dL**	48 (40-57)	52 (46-61)	0.019
**LDL-C, mg/dL**	112 (100-127)	117 (101-130)	0.511
**MetS**	27 (43.5)	7 (13.2)	0.001

Results are expressed as percentage (%) or median (IQR, 25th-75th percentile). AAV: antineutrophil cytoplasmic antibody-associated vasculitis; AMI: acute myocardial infarction; BMI: body mass index; CHF: congestive heart failure; CTR: control group; CVD: cardiovascular disease; HDL-C: high-density lipoprotein cholesterol; IQR: interquartile range; LDL-C: low-density lipoprotein cholesterol; MetS: metabolic syndrome.

Comorbidities were generally comparable between groups, except for a higher prevalence of arterial hypertension, diabetes mellitus, and dyslipidemia among patients. No cases of ischemic stroke, AMI, or CHF were reported in the CTR. In contrast, among patients with AAV, two cases of AMI (3.2%), one ischemic stroke (1.6%), and one episode of CHF (1.6%) were recorded. A positive family history of CVD was more frequent in patients than in controls (22.6% vs 7.5%; p = 0.038).

Laboratory analysis showed higher fasting glucose levels and lower total cholesterol levels in patients compared with controls. Triglyceride and LDL-C levels were similar between groups, whereas HDL-C levels were lower in the AAV group.

In multivariable analysis, both a family history of CVD (OR, 3.81; 95%CI, 1.09-12.34; p = 0.036) and MetS (OR, 4.52; 95%CI, 1.66-12.27; p = 0.003) remained independently associated with AAV.

Among patients with AAV, 27 had MetS and 35 did not. Subtype of AAV (i.e., GPA, EGPA, or MPA), overall BMI, BF percentage, incidence of cardiovascular events, physical inactivity, hypothyroidism, diabetes mellitus, family history of CVD, and total cholesterol and LDL-C levels were similar between patients with and without MetS. In univariable analysis, patients with MetS had greater waist circumference, a higher prevalence of male sex, hypertension, and dyslipidemia as well as higher fasting glucose and triglyceride levels and lower HDL-C (Table 2). Disease duration, as reflected by follow-up time in Table 2, did not differ significantly between groups.

**Table 2:** Demographic, clinical, lifestyle, comorbidity, disease characteristics, and laboratory data of patients with AAV according to MetS status

Variables	AAV with MetS (n = 27)	AAV without MetS (n = 35)	p-value
**GPA**	19 (70.4)	23 (65.7)	0.697
**EGPA**	5 (18.5)	10 (28.6)	0.359
**MPA**	3 (11.1)	2 (5.7)	0.645
**Current age, years**	55.0 (50.5-64.5)	60.0 (43.0-65.5)	0.042
**Age at diagnosis, years**	45.0 (37.5-52.8)	47.0 (32.0-57.5)	0.828
**Follow-up, months**	67.0 (21.5-135.5)	49.0 (21.0-91.5)	0.575
**Sex (male)**	19 (70.4)	9 (25.7)	0.001
**Ethnicity (White)**	21 (77.8)	32 (91.4)	0.160
**BMI, kg/m^2^**	29.7 (26.7-32.8)	26.2 (22.6-32.4)	0.042
**Waist circumference, cm**	104.0 (97.0-110.0)	94.0 (84.5-103.0)	0.013
**BF, %**	34.1 (28.5-39.2)	29.9 (24.1-34.2)	0.070
**Hypertension**	21 (77.8)	15 (42.9)	0.001
**Diabetes mellitus**	9 (33.3)	6 (17.1)	0.140
**Dyslipidemia**	27 (100)	11 (31.4)	< 0.001
**Ischemic stroke**	0	1 (2.9)	> 0.999
**AMI**	1 (3.7)	1 (2.9)	> 0.999
**CHF**	0	1 (2.9)	> 0.999
**Hypothyroidism**	2 (7.4)	5 (14.3)	0.455
**Family history of CVD**	7 (25.9)	9 (25.7)	0.984
**HAQ**	0.5 (0.0-0.75)	0.3 (0.0-1.6)	0.520
**Physical inactivity**	11 (40.7)	12 (34.3)	0.602
**Fasting blood glucose, mg/dL**	97 (87-106)	89 (82-95)	0.008
**Triglycerides, mg/dL**	106 (89-174)	97 (66-107)	0.027
**Total cholesterol, mg/dL**	171 (161-196)	182 (163-204)	0.489
**HDL-C, mg/dL**	40 (33-47)	53 (47-63)	< 0.001
**LDL-C, mg/dL**	113 (101-127)	106 (85-125)	0.438
**Active disease**	11 (40.7)	12 (34.3)	0.602
**Prednisone use**	8 (29.6)	13 (37.1)	0.535
**Prednisone dose, mg/day**	7.5 (5.0-20.0)	10.0 (5.0-20.0)	0.654
**IS/IB therapy**	21 (77.8)	27 (77.1)	0.953
**Azathioprine**	8 (29.6)	15 (42.9)	0.285
**Methotrexate**	6 (22.2)	7 (20.0)	0.831
**Mycophenolate mofetil**	8 (29.6)	3 (8.6)	0.045
**Cyclophosphamide**	2 (7.4)	0	0.186
**Rituximab**	5 (18.5)	11 (31.4)	0.294

Results are expressed as percentage (%) or median (IQR, 25th-75th percentile). AAV: antineutrophil cytoplasmic antibody-associated vasculitis; AMI: acute myocardial infarction; BF: body fat; BMI: body mass index; CHF: congestive heart failure; CVD: cardiovascular disease; EGPA: eosinophilic granulomatosis with polyangiitis; GPA: granulomatosis with polyangiitis; HAQ: Health Assessment Questionnaire; HDL-C: high-density lipoprotein cholesterol; IQR: interquartile range; IS/IB: immunosuppressive/immunobiologic; LDL-C: low-density lipoprotein cholesterol; MetS: metabolic syndrome; MPA: microscopic polyangiitis.

Disease activity and the use of prednisone (including median dose), immunosuppressive agents, or biologic therapies were similar between groups. However, mycophenolate mofetil was used more frequently among patients with MetS (p = 0.045).

In multivariable analysis, only male sex (OR, 11.43; 95%CI, 2.81-46.52; p = 0.001) and higher BMI (OR, 1.13; 95%CI, 1.02-1.25; p = 0.022) remained independently associated with the presence of MetS in patients with AAV.

## Discussion

This study demonstrated a high prevalence of MetS among patients with AAV, along with an independent association between AAV, MetS, and a family history of CVD (Central Illustration). In multivariable analysis restricted to patients with AAV, MetS was independently associated with male sex and higher BMI. Among patients with AAV, younger age and higher BMI were independently associated with MetS. A key strength of this study is the use of the most recent ACR/EULAR AAV classification criteria, which enhances diagnostic accuracy and cohort homogeneity.^8–10^ In addition, this work provides a detailed clinical and metabolic characterization of a single-center cohort of consecutive patients with AAV under regular follow-up by using a contemporary definition of MetS.

A high prevalence of MetS has been reported across systemic autoimmune rheumatic diseases, including systemic lupus erythematosus, systemic sclerosis, rheumatoid arthritis, spondyloarthritis, and autoimmune myopathies.^18–23^ In AAV, previous studies have reported prevalence rates ranging from 43% to 51.4%.^4–7^ Our findings are consistent with those of Petermann Smits et al.^4^ and Lee et al.^6^ The lower prevalence observed among controls may reflect differences in population characteristics or methodology. Most prior studies in AAV were retrospective,^4,6,7^ and the only cross-sectional study included a small sample.^5^ Earlier studies also relied on European Medicines Agency or Chapel Hill criteria, whereas the present study applied the updated ACR/EULAR definitions.^8–10^

In contrast to prior reports identifying older age as a classical risk factor for MetS in the general population, patients with MetS in this cohort were slightly younger than those without MetS. This finding may reflect earlier and more intensive exposure to glucocorticoids and immunosuppressive therapy in younger patients as well as survival and referral biases, as described in other vasculitides such as Takayasu arteritis.^24^ Younger patients with more severe or active disease may be more frequently followed at a tertiary center, whereas older patients with a greater cardiometabolic burden and prior cardiovascular events may be underrepresented in this cross-sectional sample. Most patients with MetS were male, consistent with Park et al.^7^ and with recent data indicating a rising prevalence of MetS among younger men in the general population.^25,26^

Higher BMI was independently associated with MetS, consistent with previous findings.^6^ Although BMI did not differ between patients with AAV and controls, waist circumference was higher in patients, particularly among those with MetS, highlighting central adiposity. The combination of similar BMI and higher waist circumference in patients with AAV suggests a predominance of central (abdominal) obesity, which is more strongly associated with cardiometabolic risk than generalized obesity. BF percentage did not differ between groups; adipometry was considered appropriate given its feasibility and the low prevalence of obesity.

Cardiovascular risk factors were more frequent among patients with AAV, particularly among those with MetS. Hypertension and diabetes mellitus were more common in patients with AAV than in controls, whereas triglyceride levels were higher in patients with MetS but similar between AAV and controls. Total cholesterol and HDL-C levels were lower in patients with AAV, consistent with inflammation-related lipid changes.^6^ LDL-C levels did not differ, diverging from some previous reports.^6^ Fasting glucose levels were elevated in both patients with AAV and those with MetS. A family history of CVD was more frequent among patients with AAV, whereas physical inactivity and HAQ scores were similar across groups. In multivariable analysis including both patients and controls, MetS and a positive family history of CVD remained independently associated with AAV, underscoring the unfavorable cardiometabolic profile of this cohort.

No association was observed between MetS and subtype of AAV,^5,6^ and pharmacologic treatment was largely comparable between groups, except for the more frequent use of mycophenolate mofetil among patients with MetS. This unexpected finding should be interpreted with caution. Mycophenolate mofetil is often selected for patients with specific organ involvement or pre-existing comorbidities that discourage the use of alternative immunosuppressive agents. Therefore, the higher frequency of mycophenolate use among patients with MetS likely reflects confounding by indication and physician treatment preferences rather than a direct causal effect of the drug on metabolic parameters. To the best of our knowledge, there is no robust evidence supporting a diabetogenic or dyslipidemic effect of mycophenolate mofetil, and further studies are needed to clarify this association.

This study has several limitations. First, its cross-sectional design precludes causal inference and does not allow determination of whether MetS preceded AAV onset or developed as a consequence of the disease and its treatment. Second, this is a single-center study based on a convenience sample of consecutive prevalent cases under regular follow-up at a tertiary referral Vasculitis Unit, which inherently introduces selection bias and limits generalizability. Both the AAV cohort and the CTR, recruited from hospital staff and patient companions, are unlikely to be representative of the broader AAV or general populations. Therefore, the findings should be interpreted as descriptive of this specific clinical cohort rather than as population-level estimates. Third, the relatively small sample size reduces statistical power and limits the inclusion of multiple clinically relevant confounders (such as detailed organ involvement and disease duration) in multivariable models. Fourth, although current prednisone use and dose were systematically recorded, detailed information on lifetime cumulative exposure to glucocorticoids, particularly before referral to our center, was not consistently available, which limits the assessment of its impact on MetS components. Finally, incident cardiovascular events after the diagnosis of MetS were not evaluated, as longitudinal follow-up was beyond the scope of this cross-sectional analysis.

Taken together, these limitations indicate that causal or strong epidemiological inferences should be made with caution. Prospective, longitudinal, multicenter studies with larger samples and systematic collection of cumulative treatment exposures and disease-related variables are needed to clarify the temporal relationship between AAV, MetS, and cardiovascular outcomes, including the incidence of new cardiovascular events after the diagnosis of MetS in this population.

## Conclusions

MetS is highly prevalent among patients with AAV in this single-center convenience cohort and is associated with male sex and higher BMI, contributing to an unfavorable cardiovascular risk profile. These findings highlight the need for systematic screening and aggressive management of MetS components in patients with AAV, alongside optimal control of vasculitis activity. Early identification and treatment of cardiometabolic risk factors, combined with lifestyle interventions, may reduce cardiovascular morbidity and improve long-term outcomes, although this hypothesis requires confirmation in prospective longitudinal studies.

## Data Availability

All datasets supporting the results of this study are available upon request from the corresponding author
